# Growth and Characteristics of Two Different *Epichloë sinensis* Strains Under Different Cultures

**DOI:** 10.3389/fmicb.2021.726935

**Published:** 2021-09-17

**Authors:** Yang Luo, Pei Tian

**Affiliations:** State Key Laboratory of Grassland Agro-ecosystems, Key Laboratory of Grassland Livestock Industry Innovation, Ministry of Agriculture and Rural Affairs, College of Pastoral Agriculture Science and Technology, Lanzhou University, Lanzhou, China

**Keywords:** *Epichloë sinensis*, potato dextrose agar, potato dextrose broth, exogenous additives, growth, characteristics

## Abstract

In the present study, two *Epichloë sinensis* endophyte strains isolated from different *Festuca sinensis* ecotypes were inoculated on potato dextrose agar (PDA) and potato dextrose broth (PDB) media with or without (control) exogenous additives. After 4weeks of growth, the growth (colony diameter, hyphal diameter, and mycelial biomass) and other characteristics (pH and antioxidant capacity of culture filtrate, mycelial ion contents, and hormone contents) were measured. The results showed that the culture conditions had significant effects (*p*<0.05) on the hyphal diameter, mycelial biomass, and hormone content of the two strains. The mycelial biomass of the two strains in PDB was significantly higher (*p*<0.05) than that on PDA. Except for strain 1 with indole-3-acetic acid (IAA) treatment and strain 84F with control and VB_1_ treatments, the hyphal diameter of the two strains in PDB under the other treatments was significantly higher (*p*<0.05) than that on PDA. In most cases, the IAA, cytokinins (CTK), abscisic acid (ABA), and gibberlic acid (GA) contents in the mycelia on PDA of the two strains were significantly higher (*p*<0.05) than those in PDB. The two *E. sinensis* strains exhibited significantly different performances (*p*<0.05) under the five treatments. The indices, including colony diameter, mycelial biomass, scavenging ability of superoxide anion radicals and hydroxyl radicals, pH of culture filtrate, ion contents, hyphal diameter, and IAA, CTK, GA, and ABA contents were significantly different (*p*<0.05) between the two strains, although the performance was inconsistent. Exogenous additives had significant effects (*p<*0.05) on the performance of the two *E. sinensis* strains. Indole-3-acetic acid and VB_1_ treatments significantly promoted (*p<*0.05) the growth of the two strains on both PDA and PDB. Indole-3-acetic acid treatment also significantly increased the hyphal diameters of the two strains in PDB (*p<*0.05). Indole-3-acetic acid and VB_1_ treatments significantly reduced (*p<*0.05) the antioxidant ability of these two strains in PDB. NaCl and ZnCl_2_ treatments had significant inhibitory effects (*p<*0.05) on fungal growth and promotion effects on the antioxidant ability of the two strains. The treatments also had significant effects (*p<*0.05) on hyphal diameters and ion and hormone contents, although the effects varied with different indices.

## Introduction

The grass endophytes live asymptomatically and internally within host plant tissues without causing visible damage to the plant (Siegel et al., 1987). Fungal hyphae grow in intercellular spaces in mostly leaf sheaths, culms, and seeds, while strains of *Epichloë* can be transmitted either vertically *via* seeds or horizontally *via* spores in stromata (Faeth et al., 2010). Fungal endophytes exhibit a variety of interactions with the host plant (Hume et al., 2016). The grass provides fungal endophytes with nutrients and space for growth, and in turn, fungal endophytes confer many benefits to grass hosts, including persistence/fitness, resistance/deterrence to insects, drought and salinity tolerance, and resistance to nematodes and fungal pathogens (Gundel et al., 2013; Vázquez-de-Aldana et al., 2013; Xia et al., 2018; Song et al., 2015a). This is attributed to the production of abundant and diverse secondary metabolites, as observed in a host and pure culture (Song et al., 2015b). Also, a diverse array of secondary metabolites isolated from *Epichloë* endophytes and symbionts is a rich source for developing new pesticides and drugs. However, some toxic alkaloids produced through the symbiosis also impair animal performance, which has significant economic consequences for the pastoral agricultural sectors (Schardl et al., 2012, 2013).

At present, studies on the biology and physiology of endophytes *in vitro* are mainly focused on the effects of media, conditions including temperature and pH, and carbon and nitrogen sources. For example, several studies suggested that the optimal growth conditions for *E. coenophiala* are 25°C and pH 9, whereas the optimal growth conditions of *E. gansuensis* are 25°C and pH 7 (Li et al., 2008). Latch et al. (1984) found that 25°C is the optimal temperature for the growth of *Epichloë festucae* var. *lolii*, *Epichloë coenophialum*, and *Epichloë typhina*. The optimal growth conditions for *E. festucae* are 25°C and pH 7–9 or 5 (Li et al., 2008; Ma, 2009), and the optimal growth conditions for *Epichloë bromicola* are 25°C and pH 5.09–6.10 or 9 (Zhang, 2013; Chen et al., 2016). Several studies have selected optimal nutrition resources for endophyte growth. Mannitol and tryptone are optimal carbon and nitrogen sources for *E. coenophiala* (Kulkarni and Nielsen, 1986; Pope and Hill, 1991), and maltose and tryptone are optimal carbon and nitrogen sources for *E. bromicola* (Chen et al., 2016). Lactose and casein are the optimal carbon and nitrogen sources for *E. festucae* (Li et al., 2008). Ferguson et al. found that the best nitrogen sources for the mycelial growth of *E. coenophiala* are proline and potassium nitrate (Ferguson et al., 1993). The growth of *E. festucae* is significantly improved by cellulase, tryptone, casein peptone, proline, and peptone (Ma, 2009).

*Festuca sinensis* is native to the cool semi-arid regions of China and is an important perennial cool-season bunchgrass involved in grassland establishment and ecological management programs in the Qinghai-Tibetan plateau in China (Tian et al., 2015; Zhou et al., 2015a; Wang et al., 2016). It is frequently symbiotic with *Epichloë* endophytes (Nan, 1996a). This endophyte has been isolated and identified through morphology with colony, texture, conidia, conidiophores, and its phylogeny studied through housekeeping genes. These evaluations confirmed that the endophyte that establishes symbiosis with *F. sinensis* is the new species, *Epichloë sinensis* (Tian et al., 2020). This species needs further and comprehensive studies to fully understand its characteristics and provides more reference for utilization. Previous studies have indicated that *E. sinensis* isolated from different host ecotypes performed morphological diversity which had different growth rates (Wang, 2019). Jin et al. (2009) showed that *E. sinensis* grows at 10–30°C and stop growing at 5°C and 35°C. The optimal growth conditions for *E. sinensis* are 25°C and pH 7–9, and the optimal carbon and nitrogen sources are mannitol and yeast extract. The ability to use carbon and nitrogen nutrients is different for different endophyte strains. Wang (2019) found that plant growth regulators [PGRs; VB_1_, VB_5_, VB_9_, Indole-3-acetic acid (IAA), gibberellin (GA_3_), and KT-30] promote *E. sinensis* strain growth, whereas ions (Na^+^, Cd^2+^, Cr^6+^, and Zn^2+^) inhibit their growth although the effects depend on additives and concentrations. These studies have mainly focused on the growth of *E. sinensis* under different temperatures, pH, carbon, nitrogen sources, vitamins, and heavy metals. However, little is known about the biological and physiological characteristics of *E. sinensis* in different media.

Production of fungal mycelium with high levels of secondary metabolites is critical for endophyte utilization. Compared with PDB culture for mycelium production, potato dextrose agar (PDA) culture is usually used to production (You et al., 2012), but PDA medium is not well for short-term culture to produce fungal mycelium. In addition, biomass and the biological activity of the mycelium of *Epichloë* endophytes have never been compared as between PDA and PDB culture.

In the present study, we selected two *E. sinensis* endophyte strains (strain ID 1, 84F) which were isolated from different *F. sinensis* ecotypes distributed in Gansu Province (1) and Qinghai Province (84F), China. The biological and physiological characteristics of these two strains on PDA and in PDB with different additives were measured. The objectives of this study were to: (1) compare the biological and physiological characteristics of these two *E. sinensis* strains grown in various media with exogenous additives; (2) clarify the response mechanisms of these two strains to different additives; and (3) explore the optimal enrichment conditions for *E. sinensis* mycelia.

## Materials and Methods

### Biological Materials

*Epichloë sinensis* strain 1 isolated from wild *F. sinensis* seeds in Xiahe County, Gansu Province, and strain 84F isolated from wild *F. sinensis* seeds in Ping’an County, Qinghai Province, were provided by our research groups (Kuang, 2016) and preserved in the Mycological Herbarium of Lanzhou University, China. The hyphae were transferred onto fresh PDA plates (*D*=90mm) and incubated in the dark at 25±1°C for 4weeks before the experiment.

### Experimental Design

Exogenous additives, including Vitamin B_1_ (VB_1_), IAA, sodium chloride (NaCl), and zinc chloride (ZnCl_2_), were dissolved in sterile water to make 30g/L, 20g/L, 23376g/L, and 750g/L concentrated solutions; 10μl or 100μl of each concentrated solution was added to 1L sterilized PDA or 100ml sterilized PDB, respectively, to reach final concentrations of 30mg/L, 20mg/L, 0.4mol/L, and 750mg/L, respectively. potato dextrose agar and PDB without additives were prepared as controls. A 6-mm-diameter inoculum plug of each fungus was inoculated on cellophane-overlaid PDA (*D*=90mm) containing different additives and incubated in the dark at 25±1°C for 4weeks. Each fungus had four replicates per treatment. Three 6-mm-diameter inoculum plugs of each fungus were also inoculated into PDB (100ml in 250ml flask). Each fungus had four replicates per treatment. All flasks were incubated at 25±1°C with 145rpm for 4weeks.

### Experimental Evaluations

#### Colony Diameter on PDA

The colony diameter on PDA was measured weekly during the first 4weeks of growth.

#### Hypha Diameter

Pieces of adhesive tape were gently pressed over actively growing margins of colonies on PDA to collect the mycelium. The tapes were aseptically placed on sterile glass slides for observation using an Olympus optical microscope (BX51 SZX12 type; Mm et al., 2012). Pieces of mycelia in PDB were placed on sterile glass slides and then gently covered with coverslips. Each hypha was measured through microscopy with at least 50 measurements to determine the average mycelial diameter.

#### Mycelial Biomass

Mycelia on PDA were harvested by gentle scraping with a scalpel. Mycelia and culture filtrate in PDB were separated by centrifugation at 4°C and 8,000rpm for 5min. The culture filtrate was stored at −20°C until further use. The mycelia were washed thoroughly with sterile water, and sterile water was drained off with sterilized filter paper. Each harvested mycelium was transferred into an Eppendorf tube and weighed.

#### The pH Value of Culture Filtrate

A pH meter (PB-20 type) was used to measure the pH value of the culture filtrate.

#### Antioxidant Activity of Culture Filtrate

The culture filtrate was centrifuged at 10,000rpm for 10min, and the supernatant was collected to measure the total antioxidant capacity (T-AOC). Total antioxidant capacity was determined with an ultraviolet-visible spectrophotometer according to the procedure of the Total Antioxidant Capacity Assay Kit (Nanjing Jiancheng Company, China). The absorbance of the mixture was measured at 520nm (Zeng et al., 2015).

The culture filtrate was evaporated under reduced pressure (8×103Pa) and extracted twice with 75% ethyl alcohol. Subsequently, the extract was centrifuged at 5,000rpm for 10min. The supernatant was evaporated under reduced pressure to obtain the final extract, which was stored at 4°C for further testing.

The scavenging ability of superoxide anion radicals and hydroxyl radicals was determined through the pyrogallol autoxidation and salicylic acid methods, respectively (Kim et al., 1995; Škerget et al., 2005).

#### Ion Content of Mycelia

The mycelia were freeze-dried and ground to determine the ion content. Na^+^, K^+^, and Ca^2+^ ions were measured using atomic absorption spectrometry (M6AA system, Thermo, United States) after mineralization in a mixture of acids (HNO_4_: HClO_4_=4:1), and the Na^+^/K^+^ ratio was calculated (Hanway and Heidel, 1952).

#### Endogenous Hormone Content

After 28days of growth, hyphae and culture filtrate were collected for GA_3_, IAA, cytokinins (CTK), and abscisic acid (ABA) content determination using enzyme-linked immunosorbent assay (Danshi biology, Shanghai, China; Weiler, 1984). The percentage of hormone content in the culture filtrate in total content was calculated by dividing the hormone content in the culture filtrate by the total content secreted by *E. sinensis*.

### Statistical Analyses

Statistical data analysis was performed with SPSS 25.0 (SPSS, Inc., Chicago, IL, United States). All averages and the standard error of the difference (*SE*) of the measurements were recorded in Excel2010. Univariate analysis of general linear models was employed to estimate the effects of single factor and their interaction on indices of *E. sinensis* strains in the present study (Supplementary Tables S1, S2, S3). Significant difference between single factor (exogenous additive, strain, and culture condition) was assessed by least significant difference tested at *p*<0.05 and generated from one-way ANOVA based on the separated dataset. Statistical significance was defined at the 95% confidence level.

## Results

### Colony Diameter

The colony diameters of the two *E. sinensis* strains were significantly different (*p*<0.05), and the colony diameter of strain 1 was always significantly higher (*p*<0.05) than that of strain 84F (Table 1).

**Table 1:** Colony diameter of *Epichloë sinensis* under different treatments for 4weeks (cm).

Treatment	Strain ID	
	1	84F
CK	2.93 ± 0.08c	1.95 ± 0.13c
Indole-3-acetic acid (IAA)	3.24 ± 0.08b	2.11 ± 0.07b
VB_1_	3.52 ± 0.06a	2.41 ± 0.11a
NaCl	2.3 ± 0.12d	1.5 ± 0.08d
ZnCl_2_	1.94 ± 0.07e	1.14 ± 0.16e
	^*^	

* *in the table indicates significant differences between different strains (p<0.05)*.

*Different lowercase letters in the table indicate significant differences between different exogenous additives (p<0.05)*.

Exogenous additives had significant effects (*p*<0.05) on the growth of the two strains. Compared with control, IAA and VB_1_ significantly promoted (*p*<0.05) the growth of strains 1 and 84F, whereas NaCl and ZnCl_2_ significantly inhibited (*p*<0.05) the growth of the two strains (Table 1).

### Hyphal Diameter

**Figure 1:**
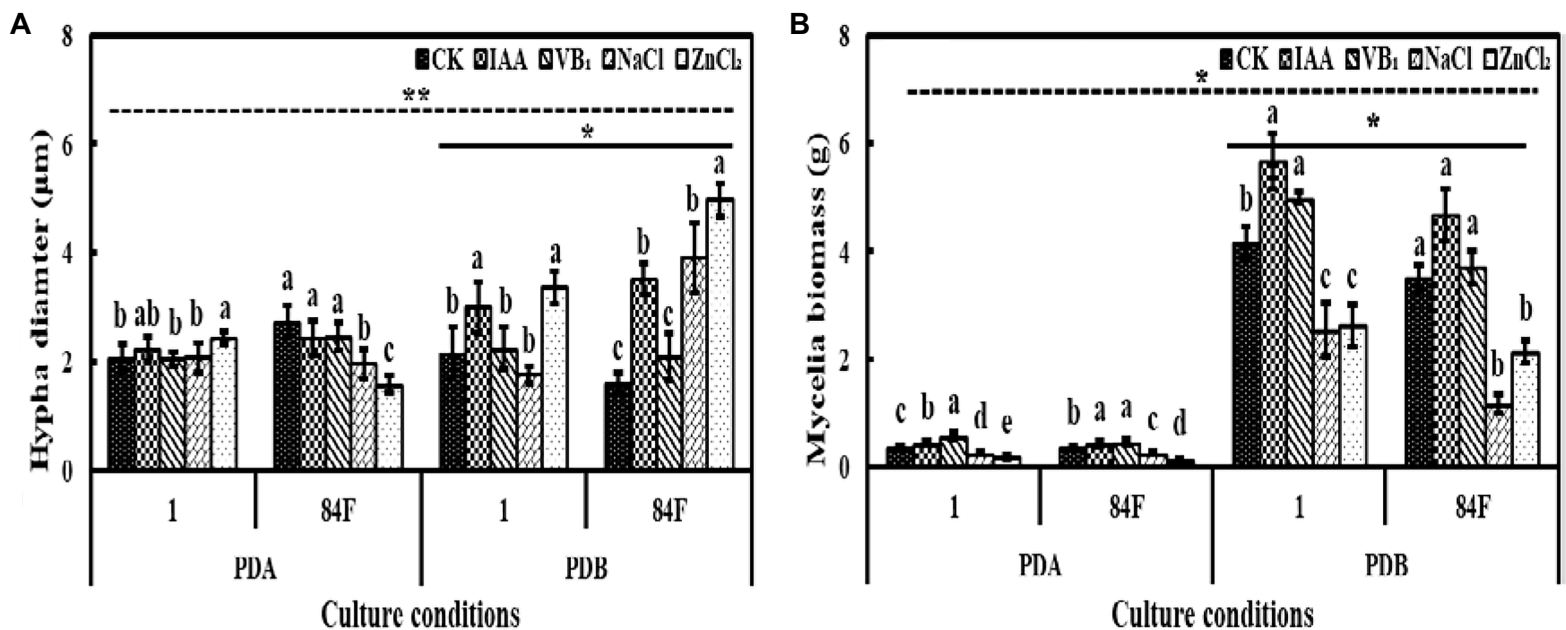
Hyphal diameter and mycelial biomass of *Epichloë sinensis* under different treatments for 4weeks. **(A)** is hyphal diameter, and **(B)** is mycelial biomass. Different lowercase letters in the figure indicate significant differences between different exogenous additives (*p*<0.05); *on the real line in the figure indicates significant differences between different strains under same culture conditions (*p*<0.05); *on the dotted line in the figure indicates significant differences between different cultures (*p*<0.05).

Culture conditions significantly affected the hyphal diameters of the two *E. sinensis* strains. The hyphal diameter of strains in PDB was significantly higher (*p*<0.05) than that on PDA (Figure 1A).

The hypha diameters of the two strains in PDB were significantly different (*p*<0.05). The hyphal diameter of strain 1 was significantly lower (*p*<0.05) than that of strain 84F (Figure 1A).

Exogenous additives had significant effects (*p*<0.05) on hyphal diameters of the two strains. The hyphal diameters of strain 1 on PDA with control, VB_1_, and NaCl treatments had no significant difference and were significantly lower (*p*<0.05) than that with ZnCl_2_ treatment. The hyphal diameters of strain 84F on PDA with control, IAA, and VB_1_ treatments had no significant difference, which was significantly higher (*p*<0.05) than that with NaCl and ZnCl_2_ treatments. The hyphal diameters of strain 1 in PDB with IAA and ZnCl_2_ were significantly higher (*p*<0.05) than those with the other three treatments; the hyphal diameters of strain 84F in PDB were significantly different and were the highest with ZnCl_2_ and the lowest with control and VB_1_ (Figure 1A).

### Mycelial Biomass

Culture conditions had significant effects (*p*<0.05) on the mycelial biomass of the two *E. sinensis* strains. The mycelial biomass of the two strains in PDB was significantly higher (*p*<0.05) than that on PDA (Figure 1B).

The mycelial biomass of the two strains in PDB was significantly different (*p*<0.05). The mycelial biomass of strain 1 was significantly higher (*p*<0.05) than that of strain 84F (Figure 1B).

Exogenous additives had significant effects (*p*<0.05) on mycelial biomass of the two strains. On PDA, the mycelial biomass of strain 1 was significantly different (*p*<0.05) under these five treatments and changed according to the following order depending on the additive: VB_1_>IAA>CK>NaCl>ZnCl_2_. The mycelial biomass of strain 84F was also significantly different (*p*<0.05) and changed according to the following order of additives: VB_1_, IAA>CK>NaCl>ZnCl_2._ In PDB with IAA and VB_1_, the mycelial biomass of strain 1 was significantly higher (*p*<0.05) than that with control treatment; however, in PDB with NaCl and ZnCl_2_, biomass of strain 1 was significantly lower (*p*<0.05) than that with control. In PDB with IAA, VB1, and control treatments, biomass of strain 84F were not significant difference (*p*>0.05), which were significantly higher (*p*<0.05) than those with NaCl and ZnCl_2_ treatments (Figure 1B).

### PH

**Figure 2:**
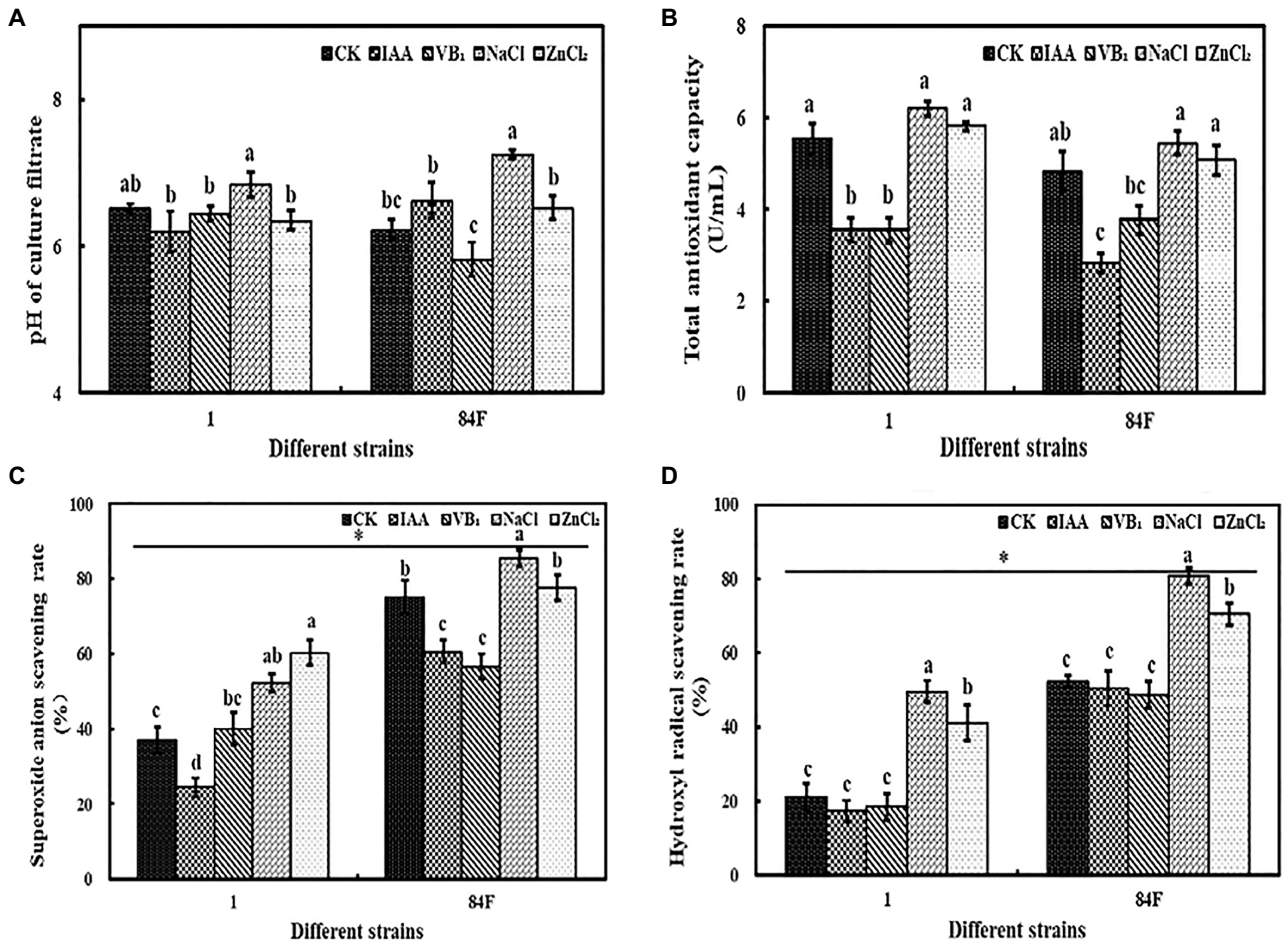
PH and antioxidant ability of *E. sinensis* under different treatments for 4weeks. **(A)** is PH, **(B)** is total antioxidant capacity of culture filtrate, **(C)** is superoxide anion radicals scavenging ability, and **(D)** is hydroxyl radical scavenging ability. The same as Figure 1.

Exogenous additives had significant effects (*p*<0.05) on the pH of the culture filtrate of the two *E. sinensis* strains. The pH of strain 1 was the highest with NaCl treatment and was significantly higher (*p*<0.05) than that with IAA, VB_1_, and ZnCl_2_ treatments. The pH of the filtrate of strain 84F with NaCl treatment was significantly higher (*p*<0.05) than that with the other treatments, and the pH of strain 84F with VB_1_ treatment was significantly lower (*p*<0.05) than that with IAA, NaCl, and ZnCl_2_ treatments (Figure 2A).

### The Antioxidant Activity

#### Total Antioxidant Activity

Exogenous additives had significant effects (*p*<0.05) on the T-AOC of the two *E. sinensis* strains. The T-AOC of strain 1 with control, NaCl, and ZnCl_2_ treatments was significantly higher (*p*<0.05) than that with IAA and VB_1_ treatments. The T-AOC of strain 84F with NaCl and ZnCl_2_ was significantly higher (*p*<0.05) than that with IAA and VB_1_ treatments (Figure 2B).

#### Superoxide Anion Radical and Hydroxyl Radical Scavenging Ability

The superoxide anion radical and hydroxyl radical scavenging abilities of the two *E. sinensis* strains were significantly different (*p*<0.05). The superoxide anion radical and hydroxyl radical scavenging abilities of strain 84F were always significantly higher (*p*<0.05) than that of strain 1 (Figures 2C,D).

Exogenous additives had significant effects (*p*<0.05) on the superoxide anion radical and hydroxyl radical scavenging abilities of the two strains. The superoxide anion radical scavenging ability of strain 1 with ZnCl_2_ treatment was significantly higher (*p*<0.05) than that with control, IAA, and VB_1_ treatments, and that with NaCl treatment was significantly higher (*p*<0.05) than that with control and IAA treatments. The superoxide anion radical scavenging ability of strain 1 was the lowest with IAA treatment. The superoxide anion radical scavenging ability of strain 84F with IAA and VB_1_ treatments was significantly lower (*p*<0.05) than that with control, NaCl, and ZnCl_2_ treatments. The hydroxyl radical scavenging ability of strains 1 and 84F with NaCl treatment was significantly higher (*p*<0.05) than that with the other four treatments, while the scavenging ability with ZnCl_2_ treatment was significantly higher (*p*<0.05) than that with control, IAA, and VB_1_ treatments (Figures 2C,D).

#### Ion Content of Mycelia

**Figure 3:**
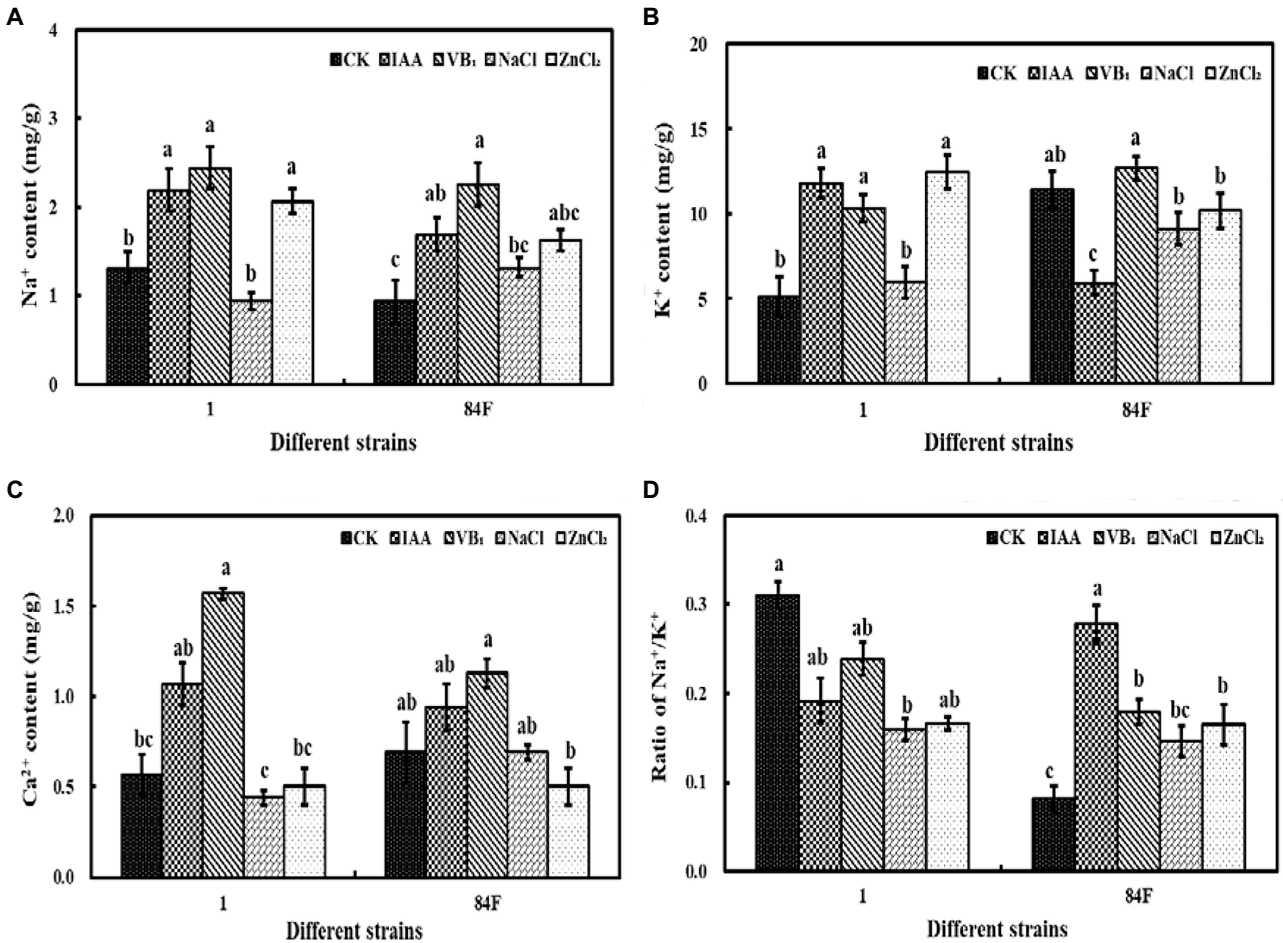
Na^+^, K^+^, and Ca^2+^ contents and ratio of Na^+^/K^+^ of mycelium of *E. sinensis* under different treatments for 4weeks. **(A)** is Na^+^ content, **(B)** is K^+^ content, **(C)** is Ca^2+^ content, and **(D)** is ratio of Na^+^/K^+^. The same as Figure 1.

Exogenous additives had significant effects (*p*<0.05) on the Na^+^, K^+^, and Ca^2+^ contents of the two *E. sinensis* strains. Na^+^ and K^+^ contents of strain 1 with IAA, VB_1_, and ZnCl_2_ were significantly higher (*p*<0.05) than those with control and NaCl treatments. The Na^+^ content of strain 84F with VB_1_ treatment was significantly higher (*p*<0.05) than that with control and NaCl treatments. The K^+^ content of strain 84F with VB_1_ treatment was significantly higher (*p*<0.05) than that with IAA, NaCl, and ZnCl_2_ treatments. The Ca^2+^ content of strain 1 with VB_1_ treatment was significantly higher (*p*<0.05) than that with control, NaCl, and ZnCl_2_ treatments. The Ca^2+^ content of strain 84F with VB_1_ treatment was significantly higher (*p*<0.05) than that with ZnCl_2_ treatment. There was no significant difference between Na^+^, K^+^, and Ca^2+^ contents in mycelia of the two strains (Figures 3A–C).

Exogenous additives had significant effects on the Na^+^/K^+^ ratio of the two strains. Na^+^/K^+^ of strain 1 with NaCl treatment was significantly lower (*p*<0.05) than that with control. Na^+^/K^+^ of strain 84F with control was significantly lower (*p*<0.05) than that of IAA, VB_1_, and ZnCl_2_ treatments, and the ratio with IAA treatment was significantly higher (*p*<0.05) than that with the other three treatments (Figure 3D).

### Hormone Content

#### CTK Content

**Figure 4:**
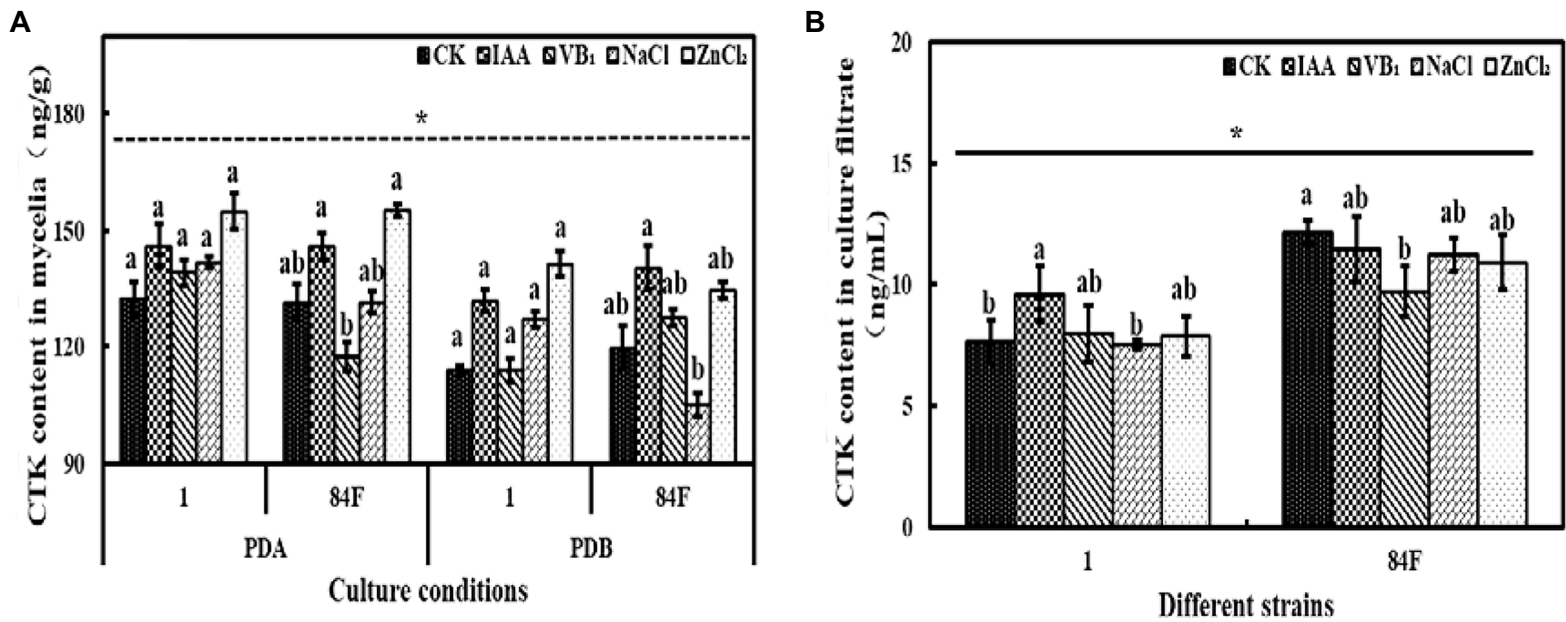
Cytokinins content of *E*. sinensis under different treatments for 4weeks. **(A)** is CTK content in mycelia, and **(B)** is CTK content in culture filtrate. The same as Figure 1.

Culture conditions had significant effects (*p*<0.05) on CTK content in mycelia of the two *E. sinensis* strains. The CTK content in mycelia of *E. sinensis* strains on PDA was significantly higher (*p*<0.05) than that in PDB (Figure 4).

Cytokinins content in mycelia of the two strains on PDA and in PDB was not significantly different (*p*<0.05). Cytokinins contents in the culture filtrate of strain 84F were significantly higher (*p*<0.05) than that of strain 1 (Figure 4).

Exogenous additives had significant effects (*p*<0.05) on CTK content on PDA and in PDB of the two strains. Cytokinins contents in mycelia of strain 1 on PDA and in PDB were not significantly different among the five treatments. The CTK contents of strain 84F on PDA with IAA and ZnCl_2_ treatments were significantly higher (*p*<0.05) than those with VB_1_ treatment. In PDB with IAA, the CTK content in mycelia of strain 84F was significantly higher (*p*<0.05) than that with NaCl treatment. Cytokinins content in the culture filtrate of strain 1 with IAA treatment was significantly higher (*p*<0.05) than that with control and NaCl treatments; CTK content in the culture filtrate of strain 84F with control treatment was significantly higher (*p*<0.05) than that with VB_1_ treatment (Figure 4).

#### ABA Content

**Figure 5:**
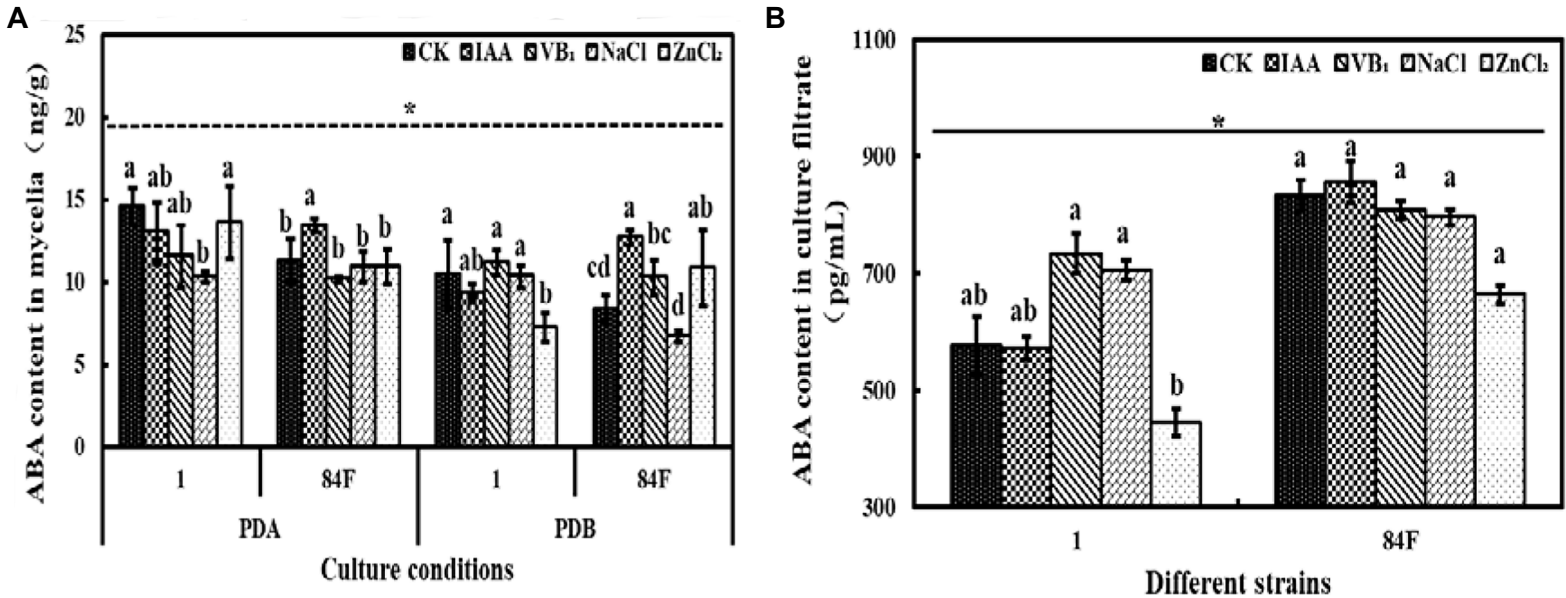
Abscisic acid content of *E*. sinensis under different treatments for 4weeks. **(A)** is ABA content in mycelia, and **(B)** is ABA content in culture filtrate. The same as Figure 1.

Culture conditions had significant effects (*p*<0.05) on CTK content in mycelia of the two *E. sinensis* strains. Abscisic acid content in mycelia of *E. sinensis* strains on PDA was significantly higher (*p*<0.05) than that in PDB (Figure 5).

The ABA content in mycelia of these two strains was significantly different (*p*<0.05). In the culture filtrate, the ABA content of strain 84F was significantly higher (*p*<0.05) than that of strain 1 (Figure 5).

Exogenous additives had significant effects (*p*<0.05) on the ABA content of the two strains. On PDA, the ABA contents of strain 1 with control and ZnCl_2_ treatments were significantly higher (*p*<0.05) than those with NaCl treatment; the ABA content for strain 84F with IAA treatment was significantly higher (*p*<0.05) than that with the other four treatments. In PDB, ABA contents in the mycelia of strain 1 with control, VB_1_, and NaCl treatments were significantly higher (*p*<0.05) than those with ZnCl_2_ treatment. Abscisic acid content in the mycelia of strain 84F with IAA treatment was significantly higher (*p*<0.05) than that with control, VB_1_, and NaCl treatments. In PDB with ZnCl_2_ treatment, ABA content in mycelia of strain 84F was significantly higher (*p*<0.05) than that with control and NaCl treatments. The ABA contents in the culture filtrate of strain 1 with VB_1_ and NaCl treatments were significantly higher (*p*<0.05) than those with ZnCl_2_ treatment (Figure 5).

#### GA Content

**Figure 6:**
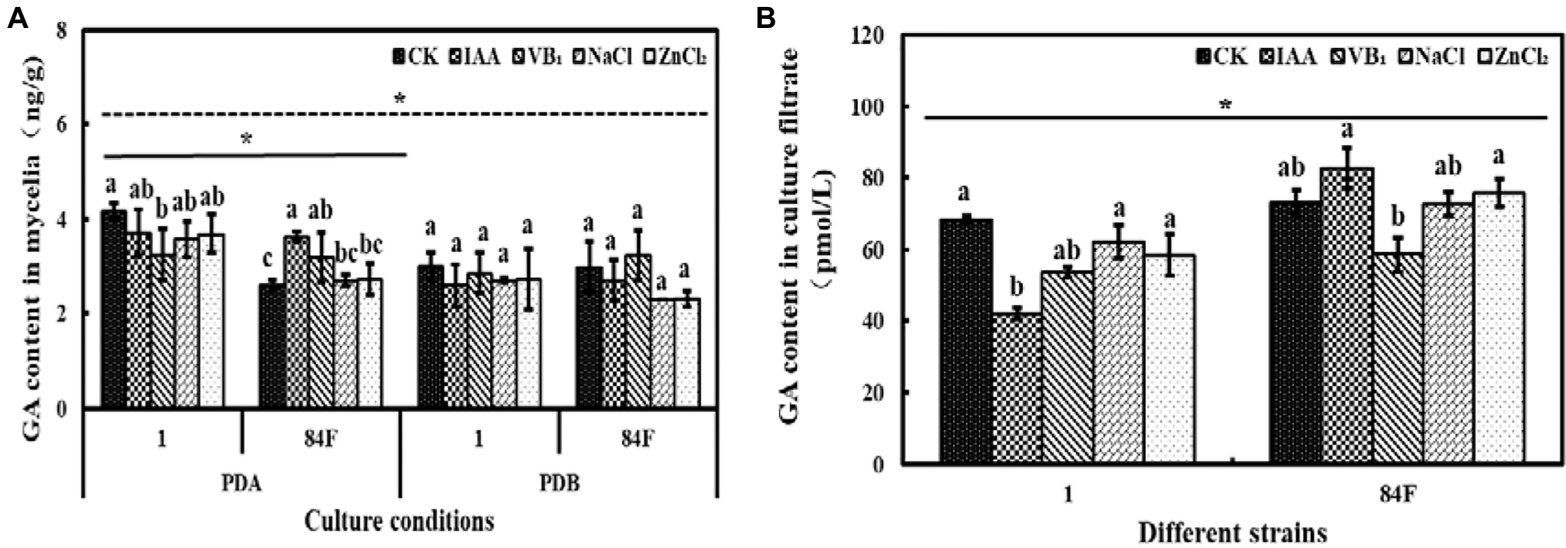
Gibberlic acid content of *E*. sinensis under different treatments for 4weeks. **(A)** is GA content in mycelia, and **(B)** is GA content in culture filtrate. The same as Figure 1.

Culture conditions had significant effects (*p*<0.05) on GA content in mycelia of the two *E. sinensis* strains. GA content in mycelia of *E. sinensis* strains on PDA was significantly higher (*p*<0.05) than that in PDB (Figure 6).

GA content in mycelia of the two strains was significantly different (*p*<0.05). On PDA, the GA content of strain 1 was significantly higher (*p*<0.05) than that of 84F. In PDB, no significant difference was observed between the two strains. In the culture filtrate, the GA content of strain 84F was significantly higher (*p*<0.05) than that of strain 1 (Figure 6).

Exogenous additives had significant effects (*p*<0.05) on the GA content of the two strains. On PDA, the GA content of strain 1 with control was significantly higher (*p*<0.05) than that with VB_1_ treatment. The GA content of strain 84F with IAA treatment was significantly higher (*p*<0.05) than that with control, NaCl, and ZnCl_2_ treatments. In PDB, there were no significant differences between GA contents in mycelia of strains 1 and 84F under the five treatments. GA content in the culture filtrate of strain 1 with IAA treatment was significantly lower (*p*<0.05) than that with control, NaCl, and ZnCl_2_ treatments; GA contents in the culture filtrate of strain 84F with IAA and ZnCl_2_ treatments were significantly higher (*p*<0.05) than those with VB_1_ treatment (Figure 6).

#### IAA Content

**Figure 7:**
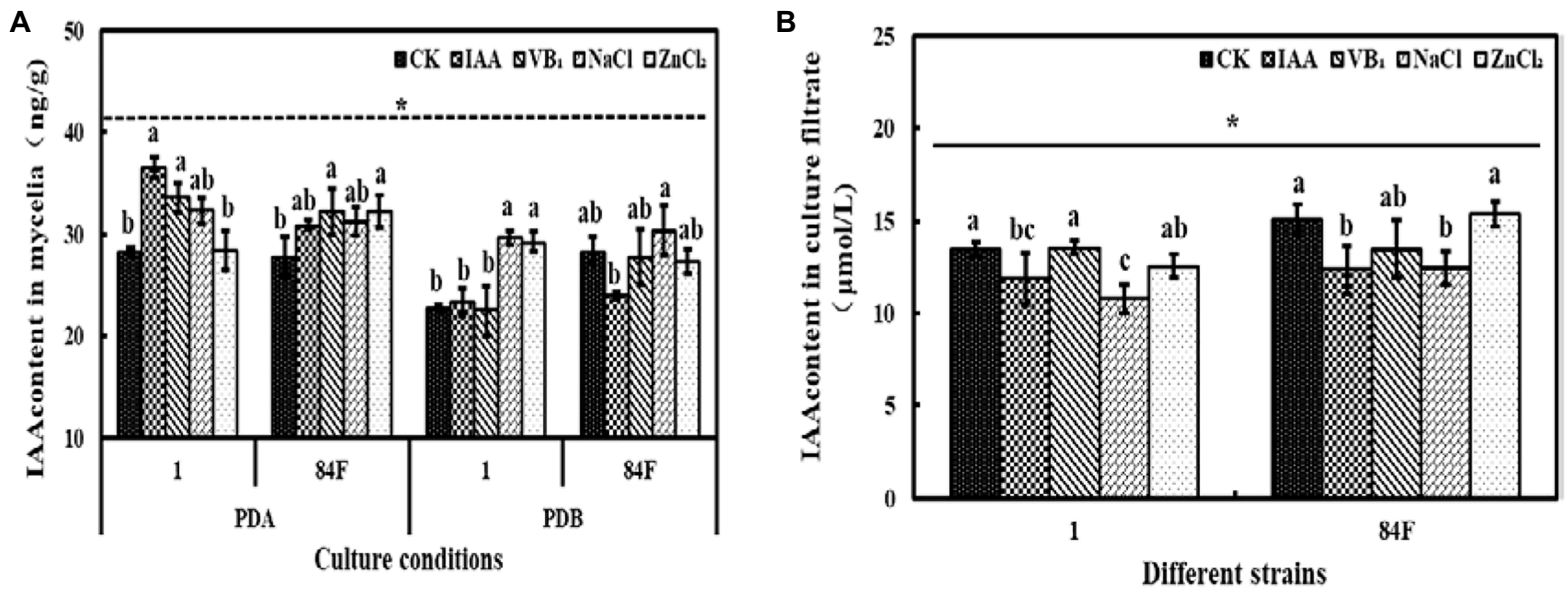
Indole-3-acetic acid content of *E*. sinensis under different treatments for 4weeks. **(A)** is IAA content in mycelia, and **(B)** is IAA content in culture filtrate. The same as Figure 1.

Culture conditions had significant effects (*p*<0.05) on IAA content in mycelia of the two *E. sinensis* strains. The IAA content in mycelia of *E. sinensis* strains on PDA was significantly higher (*p*<0.05) than that in PDB (Figure 7).

IAA content in mycelia of the two strains on PDA and in PDB was not significantly different (*p*<0.05). IAA contents in the culture filtrate of strain 84F were significantly higher (*p*<0.05) than those of strain 1 (Figure 7).

Exogenous additives had significant effects (*p*<0.05) on the IAA content of the two strains. On PDA with IAA and VB_1_ treatments, IAA contents of strain 1 were significantly higher (*p*<0.05) than those with control and ZnCl_2_ treatments. In PDB, IAA content in mycelia of strain 1 with NaCl and ZnCl_2_ treatments was significantly higher (*p*<0.05) than those with control, IAA, and VB_1_ treatments. Indole-3-acetic acid content in mycelia of strain 84F with NaCl treatment was significantly higher (*p*<0.05) than those with IAA treatment. Indole-3-acetic acid content in the culture filtrate of strain 1 with control and VB_1_ treatments was significantly higher (*p*<0.05) than those with IAA and NaCl treatments. Indole-3-acetic acid content in the culture filtrate of strain 84F with control and ZnCl_2_ treatments was significantly higher (*p*<0.05) than those with IAA and NaCl treatments (Figure 7).

#### The Percentage of Hormone Content in the Culture Filtrate in Total Content Secreted

The percentages of CTK, ABA, and GA contents in the culture filtrate in total contents secreted by these two strains were significantly different (*p*<0.05). The percentage of strain 84F was significantly higher (*p*<0.05) than that of strain 1 (Table 2).

**Table 2:** Percentage of hormone content in the culture filtrate in total content (%).

Treatment	Cytokinins (CTK)		Abscisic acid (ABA)		Gibberlic acid (GA)		Indole-3-acetic acid (IAA)	
	1	84F	1	84F	1	84F	1	84F
CK	40.17a	50.41a	36.03a	49.26ab	44.23a	46.28b	50.86a	48.24a
IAA	42.31a	44.80b	37.37a	40.17ab	36.23a	51.59a	47.03b	47.38a
VB'	41.16a	43.56b	39.60a	43.89ab	38.80a	39.13a	51.41a	45.95ab
NaCl	37.38a	51.63a	40.23a	53.96a	44.21a	52.00ab	38.91d	41.77b
ZnCl'	35.75a	44.84b	37.78a	38.21b	43.14a	53.15a	42.89c	49.73a
	^*^		^*^		^*^			

* *in the table indicates significant differences between different strains (p<0.05)*.

*Different lowercase letters in the table indicate significant differences between different exogenous additives (p<0.05)*.

Exogenous additives had significant effects (*p*<0.05) on the percentage of IAA content in the culture filtrate in total content secreted by strain 1. The percentage of strain 1 with control and VB_1_ treatments was significantly higher (*p*<0.05) than that with the other four treatments; the percentage of strain 1 was significantly different (*p*<0.05) and changed according to the following order of additives: CK, VB_1_>IAA>NaCl>ZnCl_2._ Exogenous additives had significant effects (*p*<0.05) on the percentage of CTK content in the culture filtrate in total content secreted by strain 84F (*p*<0.05). The percentages of CTK content in the culture filtrate in total content secreted by strain 84F with control and NaCl treatments were significantly higher (*p*<0.05) than that with IAA, VB_1_, and ZnCl_2_ treatments. The percentage of ABA content in the culture filtrate in total content secreted by strain 84F with NaCl treatment was significantly higher (*p*<0.05) than that with ZnCl_2_ treatment and was not significantly different with control, IAA, and VB_1_ treatments. The percentage of GA content in the culture filtrate in total content secreted by strain 84F with VB_1_ treatment was significantly higher (*p*<0.05) than that with IAA, NaCl, and ZnCl_2_ treatments. The percentage of IAA content in the culture filtrate in total content secreted by strain 84F with NaCl treatment was significantly lower (*p*<0.05) than that with the other treatments (Table 2).

## Discussion

The endophyte strains isolated from different host ecotypes showed a rich diversity in morphological and growth characteristics (Wei et al., 2010). Ahlholm et al. (2002) showed that host genotypes, along with prevailing environmental conditions, influenced the genetic variation of endophytes. Endophyte strains isolated from a globally distributed collection of perennial ryegrass accessions presented rich gene diversity after evaluation with simple sequence repeat markers (van Zijll De Jong et al., 2008a, 2008b). The genetic polymorphism of grass endophytes is beneficial to select non-toxic strains that do not produce toxic alkaloids to livestock. These selected endophytes were artificially inoculated into grass and established a new grass-endophyte symbiosis that is both stress-resistant and non-toxic to domestic animals and, therefore, improves the quality of grasses and ensures animal safety (Johnson et al., 2013; Young et al., 2013). Yang et al. (2011) found differences in the culture characteristics and growth rates of various endophyte strains isolated from wild *F. sinensis* seeds from Sangke and Ganjia grasslands in the Gansu Province, China (Yang et al., 2011). Significant diversity was observed among the five *E. sinensis* endophyte strains isolated from different *F. sinensis* ecotypes on PDA with a variety of additives at different growth rates (Wang, 2019). The results of the present study support these findings and indicated that *E. sinensis* strains are biodiverse, and the growth and physiological characteristics of *E. sinensis* are affected by host ecotypes. The selection of appropriate markers for genetic polymorphism analysis of *E. sinensis* strains was conducted to reveal the relationship between their growth rate, physiological changes, and host genotype.

The endophyte growth is influenced not only by host genotype, environmental conditions but also by culture conditions (Ahlholm et al., 2010; Wei et al., 2010). We studied the growth of five *E. sinensis* endophyte strains on PDA with different additives and found that PGRs (VB_1_, VB_5_, VB_9_, IAA, GA_3_, and KT-30) promoted fungal growth, whereas ions (Na^+^, Cd^2+^, Cr^6+^, and Zn^2+^) inhibited it (Wang, 2019). The growth of four *E. sinensis* strains with 30mg/l VB_1_ and five *E. sinensis* strains with 20mg/L IAA significantly increased (*p<*0.05), whereas the five strains had the strongest resistance with 0.4mol/L Na^+^ and 750mg/L Zn^2+^. Therefore, the four different treatments which included 30mg/L VB_1_, 20mg/L IAA, 0.4mol/L NaCl, and 750mg/L ZnCl_2_ in this study were set up based on previous experiments and were initiated to determine the response mechanism of these two strains to different additives.

Numerous studies have shown that IAA and VB_1_ promote mycelial growth of fungi (Ren et al., 2007; Li et al., 2016; Luo et al., 2021). High concentrations of NaCl and ZnCl_2_ inhibited the colony diameter of *E. gansuensis* and *Penicillium chrysogenum*, respectively (Jin, 2009; Wang et al., 2020). The present results are similar to those of other studies. Numerous reports have indicated that the application of PGRs, including phytohormones, promotes cell division and enhances plant and microbe growth (Naeem et al., 2004). However, vitamins only play a regulatory role in plant metabolism to maintain normal central metabolic processes (Liao et al., 2018). In this experiment, compared with the control treatment, the promotion effect of IAA treatment on *E. sinensis* was higher than that of VB_1_, which may be related to the different effects of vitamins and plant hormones in the organism. The resistance of *E. sinensis* to Na^+^ was stronger than that of Zn^2+^, indicating that *E. sinensis* has different resistance to different metal ions.

Endophytes produce numerous antioxidant compounds that may play roles in enhancing stress tolerance (Schulz et al., 2002; Malinowski and Belesky, 2006; Yuan et al., 2010). Most abiotic (desiccation and/or rehydration, nutrient limitation, and UV radiation) and biotic stresses (pathogens and insect herbivory) induce reactive oxygen species (ROS) production (Beckett et al., 2005). Cells need appropriate systems to allow rapid removal of ROS. Previous studies have found that the optimal zinc concentration in the medium may improve *Lentinus edodes* mycelium growth and the zinc content of the *L. edodes* mycelium, and increase antioxidant enzyme activities (Gang et al., 2017). The antioxidant capacity of endophytes from *Myricaria laxiflora* was extraordinarily high under mild saline-alkali stress (Gao et al., 2016). The present study found that the two *E. sinensis* culture filtrates showed high free radical scavenging ability with NaCl and ZnCl_2_ treatments, which demonstrated that the antioxidant capacity played an important role during stress resistance of *E. sinensis*. The scavenging ability of superoxide anion radicals and hydroxyl radicals of strain 84F was higher than that of strain 1 under different treatments, which could be used to further investigate the natural antioxidants of the two *E. sinensis* strains.

Potato dextrose broth is currently widely used in the industrial production of medicinal and edible fungi because of the short fungal production cycle (Yang et al., 2000; Huang and Zhang, 2005). In the present study, the mycelial biomass and hyphae diameter in PDB were generally higher than those on PDA. These differences confirmed that PDB medium has the advantage of uniform distribution of nutrients during microbial cultivation, which is conducive to full contact and absorption of nutrients by mycelia cells, and eventually increases the production of mycelia and nutrients. Therefore, PDB can also be applied to enrich more *E. sinensis* mycelium.

Hormones are naturally occurring organic molecules that regulate plant growth and its developmental processes (Costacurta and Vanderleyden, 1995; Hoyerova et al., 2006; Berger et al., 2007) and present in a wide variety of organisms, including fungi (Battista et al., 1990; Zhang et al., 1999; Yue et al., 2000; Yuan et al., 2004; Nambara and Marionpoll, 2005; Hartung, 2010; Spichal, 2012; Ban, 2013). In the present study, IAA, CTK, IAA, and GA_3_ in mycelia and fermentation of two *E. sinensis* strains under five treatments were quantified, suggesting that the *E. sinensis* strain produces growth-promoting hormones (Lee, 1990; Abd, 1997; Deotale et al., 1998; Klingler et al., 2011; Chanclud et al., 2016). Previous studies have found *Epichloë* endophyte change hormones to improve host stress tolerance (Battista et al., 1990; Saikkonen et al., 2004; Xia et al., 2018). In this study, hormones were detected in both mycelia and fermentation, indicating that the hormone concentration increased in E+ plants may be also adjusted by endophyte. In most case, the percentages of hormone contents in the culture filtrate in total contents secreted by *E. sinensis* were about 50%. These results provide a basis for the development and utilization of *E. sinensis* strains. In addition, host ecotypes, culture conditions, and exogenous additives affected the hormone content of the strains. However, these effects were not consistent.

Maintaining a constant intracellular K^+^ and Na^+^ balance is essential for metabolic processes in cells (Zhu, 2003). Restriction of the transport of Na^+^ and increase in the K^+^ concentration to ensure a high cytosolic K^+^/Na^+^ ratio are very important for cells to tolerate stress (Berthomieu et al., 2003; Cuin et al., 2003). Ca^2+^ is essential for selective ion transport mechanisms and for the maintenance of K^+^ influx and Na^+^/K^+^ selectivity. This study is one of the few studies on ion response of *E. sinensis* in various media with exogenous additives. Our results showed exogenous additives had effects on Na^+^, K^+^, and Ca^2+^ contents and the Na^+^/K^+^ ratio of these two strains and the effects on two strains were inconsistent. This finding suggested *E. sinensis* may adapt to changing environment by regulating ion contents. Compared with other treatments, Na^+^ content in mycelia of the two strains was not significantly different or significantly reduced with the NaCl treatment. This may be because organisms selectively absorb or transport ions to reduce their stress damage and maintain normal physiological metabolism. Therefore, exogenously added Na^+^ might remain in the culture filtrate. Ion contents on PDA and in the culture filtrate should be further studied to understand the resistance mechanism of *E. sinensis* strains.

## Conclusion

Our results demonstrate that *E. sinensis* strains isolated from different host ecotypes showed a rich diversity in physiology and biochemistry characteristics in various media with exogenous additives. *E. sinensis* strain might adapt to environmental changes by changing its antioxidant capacity or intracellular ion content. Additionally, PDB culture with IAA and VB_1_ additives was the optimal enrichment conditions for *E. sinensis* mycelia and the two *E. sinensis* strains produced IAA, ABA, GA, and CTK to a certain level under different treatments. Collectively, our findings provide a theoretical basis for fully understanding *E. sinensis* and obtain more reference for utilization.

## Data Availability Statement

The raw data supporting the conclusions of this article will be made available by the authors, without undue reservation.

## Author Contributions

PT designed the experiments. YL did the experiment and analysis. All authors wrote the manuscript, contributed to the article, and approved the submitted version.

## Funding

The research reported here was funded by the National Nature Science Foundation of China (31971768; 32061123004), China Agriculture Research System (CARS-22 Green Manue), and Lanzhou University enterprise-funded project [(19)0439].

## Conflict of Interest

The authors declare that the research was conducted in the absence of any commercial or financial relationships that could be construed as a potential conflict of interest.

## Publisher’s Note

All claims expressed in this article are solely those of the authors and do not necessarily represent those of their affiliated organizations, or those of the publisher, the editors and the reviewers. Any product that may be evaluated in this article, or claim that may be made by its manufacturer, is not guaranteed or endorsed by the publisher.

## Supplementary Material

**Supplementary Table S1** | Univariate analysis of general linear models was employed to estimate the effects of single factor and their interaction on hypha diameter and mycelia biomass of *Epichloë sinensis* strains in the present study.

**Supplementary Table 2** | Univariate analysis of general linear models was employed to estimate the effects of single factor and their interaction on cytokinins, abscisic acid, gibberlic acis, and indole-3-acetic acid contents of *E. sinensis* strains in the present study.

**Supplementary Table 3** | Univariate analysis of general linear models was employed to estimate the effects of single factor and their interaction on pH, total antioxidant capacity, scavenging abilities of superoxide anion radical and hydroxyl radical, and ion contents of *E. sinensis* strains in the present study.

## References

[ref1] Abd E. I. (1997). Effect of phosphorus, boron, GA_3_ and their interactions on growth, flowering, pod setting, abscission and both green pod and seed yields of broad been (*Viciafaba* L.) plant. Alexandria J. Agrc. Res. 42, 311–332.

[ref2] Ahlholm J. U. Helander M. Henriksson J. Metzler M. Saikkonen K. (2002). Environmental conditions and host genotype direct genetic diversity of *Venturia ditricha*, a fungal endophyte of birth trees. Evolution 56, 1566–1573. doi: 10.1111/j.0014-3820.2002.tb01468.x, PMID: 12353749

[ref3] Ahlholm J. U. Helander M. Henriksson J. Metzler M. Saikkonen K. (2010). Environmental conditions and host genotype direct genetic diversity of *Venturia ditricha*, a fungal endophyte of birch trees. Evolution 56, 1566–1573. doi: 10.1111/j.0014-3820.2002.tb01468.x, PMID: 12353749

[ref4] Ban Y. H. (2013). Mechanisms of dark septate endophyte isolated from Pb-Zn mine improving plant lead tolerance. dissertation/master’s thesis. Yangling: Xianyang. Northwest Agriculture and Forestry University, China.

[ref5] Battista J. P. D. Bacon C. W. Severson R. Plattner R. D. Bouton J. H. (1990). Indole acetic acid production by the fungal endophyte of tall fescue. Agron. J. 82, 878–880. doi: 10.2134/agronj1990.00021962008200050006x

[ref002] Beckett R. P. Minibayeva F. V. Laufer Z. (2005). Extracellular reactive oxygen species production by lichens. Lichenologist 37, 397–40. doi: 10.1017/S0024282905014921

[ref6] Berger S. Sinha A. K. Roitsch T. (2007). Plant physiology meets phytopathology: plant primary metabolism and plant-pathogen interactions. J. Exp. Bot. 58, 4019–4026. doi: 10.1093/jxb/erm298, PMID: 18182420

[ref7] Berthomieu P. Conéjéro G. Nublat A. Brackenbury W. J. Lambert C. Savio C.. (2003). Functional analysis of *AtHKT1* in *Arabidopsis* shows that Na^+^ recirculation by the phloem is crucial for salt tolerance. EMBO J. 22, 2004–2014. doi: 10.1093/emboj/cdg207, PMID: 12727868 PMC156079

[ref8] Chanclud E. Kisiala A. Emery N. R. J. Chalvon V. Ducasse A. Romiti-Michel C.. (2016). Cytokinin production by the rice blast fungus is a pivotal requirement for full virulence. PLoS Pathog. 12:e1005457. doi: 10.1371/journal.ppat.1005457, PMID: 26900703 PMC4765853

[ref9] Chen T. X. Li C. J. Li X. Z. (2016). Biological and physiological characteristics of *Epichloё bromicola* endophyte symbiotic with *Hordeum brevisubulatum*. Pratacult Sci. 33, 1658–1664. doi: 10.11829/j.issn.1001-0629.2015-0617

[ref10] Costacurta A. Vanderleyden J. (1995). Synthesis of phytohormones by plant-associated bacteria. Crit. Rev. Microbiol. 21, 1–18. doi: 10.3109/10408419509113531, PMID: 7576148

[ref11] Cuin T. A. Miller A. J. Laurie S. A. Leigh R. A. (2003). Potassium activities in cell compartments of salt-grown barley leaves. J. Exp. Bot. 54, 657–661. doi: 10.1093/jxb/erg072, PMID: 12554708

[ref12] De Jong E. V. Dobrowolski M. P. Bannan N. R. Stewart A. V. Smith K. F. Spangenberg G. C.. (2008a). Global genetic diversity of the perennial ryegrass fungal endophyte *Neotyphodium lolii*. Crop Sci. 48, 1487–1501. doi: 10.2135/cropsci2007.11.0641

[ref13] De Jong E. V. Dobrowolski M. P. Sandford A. Smith K. F. Willocks M. J. Spangenberg G. C.. (2008b). Detection and characterisation of novel fungal endophyte genotypic variation in cultivars of perennial ryegrass *(Lolium perenne* L.). Aust. J. Agric. Res. 59, 214–221. doi: 10.1071/AR07270

[ref14] Deotale R. D. Mask V. G. Sorte N. V. Chimurkar B. S. Yerne A. Z. (1998). Effect of GA_3_ and IAA on morpho-physiological parameters of soybean. J. Soils Crops 8, 91–94.

[ref15] Faeth S. H. Helander M. L. Saikkonen K. T. (2010). Asexual Neotyphodium endophytes in a native grass reduce competitive abilities. Ecol. Lett. 7, 304–313. doi: 10.1111/j.1461-0248.2004.00578.x

[ref16] Ferguson N. H. Rice J. S. Allgood N. G. (1993). Variation in nitrogen-utilization in *Acremonium coenophialum* isolates. Appl. Environ. Microbiol. 59, 3602–3604. doi: 10.1128/aem.59.11.3602-3604.1993, PMID: 16349079 PMC182505

[ref17] Gang J. Zhang S. Liu Y. Xie B. Pang S. L. (2017). Effects of zinc on the growth and antioxidant enzymes of *lentinus edodes* mycelium. Food Ferment. Ind. 43, 146–151. doi: 10.13995/j.cnki.11-1802/ts.201706024

[ref18] Gao Y. Lei Q. Jiang W. Kong Y. S. Xue Y. H. Liu S. P. (2016). Molecular characterization and phenolic acids analysis of an endophytic fungus with high antioxidant activity. Microbiology 43, 1235–1243. doi: 10.13344/j.microbiol.china.150534

[ref19] Gundel P. E. Pérez L. I. Helander M. Saikkonen K. (2013). Symbiotically modified organisms: nontoxic fungal endophytes in grasses. Trends Plant Sci. 18, 425–432. doi: 10.1016/j.tplants.2013.03.003, PMID: 23562460

[ref20] Hanway J. J. Heidel H. (1952). Soil analysis methods as used in lowa state college soil testing laboratory. Iowa Agric. 57, 1–31.

[ref21] Hartung W. (2010). The evolution of abscisic acid (ABA) and ABA function in lower plants, fungi and lichen. Funct. Plant Biol. 37, 806–812. doi: 10.1071/FP10058

[ref22] Hoyerova K. Gaudinova A. Malbeck J. Dobrev P. I. Kocabek T. Solcova B.. (2006). Efficiency of different methods of extraction and purification of cytokinins. Phytochemistry 67, 1151–1159. doi: 10.1016/j.phytochem.2006.03.010, PMID: 16678229

[ref23] Huang Q. Zhang L. (2005). Solution properties of (1→3)-alpha-D-glucan and its sulfated derivative from *Poriacocos mycelia* via fermentation tank. Biopolymers 79, 28–38. doi: 10.1002/bip.20332, PMID: 15957178

[ref24] Hume D. E. Ryan G. D. Gibert A. Helander M. Mirlohi A. Sabzalian M. R. (2016). *Epichloë* fungal endophytes for grassland ecosystems. Sustainable Agric. Rev. 19, 233–305.

[ref25] Jin W. J. (2009). Diversity of *Neotyphodium* endophytes symbiotic with *Achnatherum inebrians*. dissertation/master’s thesis. (Lanzhou (Gansu): LanZhou University).

[ref26] Jin W. J. Li C. J. Nan Z. B. (2009). Biological and physiological characteristics of *Neotyphodium* endophyte symbiotic with *Festuca sinensis*. Mycosystema 28, 363–369.

[ref27] Johnson L. J. Bonth A. Briggs L. R. Garadus J. R. Finch S. C. Fleetwood D. J.. (2013). The exploitation of epichloae endophytes for agricultural benefit. Fungal Divers. 60, 171–188. doi: 10.1007/s13225-013-0239-4

[ref28] Kim S. J. Han D. Moon K. D. Rhee J. S. (1995). Measurement of superoxide dismutase-like activity of natural antioxidants. Biosci. Biotechnol. Biochem. 59, 822–826. doi: 10.1271/bbb.59.822, PMID: 7787296

[ref29] Klingler J. P. Batelli G. Zhu J. K. (2011). ABA receptors: the START of a new paradigm in phytohormone signalling. J. Exp. Bot. 61, 3199–3210. doi: 10.1093/jxb/erq151, PMID: 20522527 PMC3107536

[ref30] Kuang Y. (2016). Characteristics of *Epichloë* endophyte-*Festuca sinensis* symbiote. dissertation/master’s thesis. Lanzhou (Gansu): LanZhou University.

[ref31] Kulkarni R. K. Nielsen B. D. (1986). Nutritional requirements for growth of a fungus endophyte of tall fescue grass. Mycologia 78, 781–786. doi: 10.2307/3807523

[ref32] Latch G. Christensen M. J. Samuels G. J. (1984). Five endophytes of *Lolium* and *Festuca* in New Zealand. Mycotaxon 20, 338–342. doi: 10.1192/bjp.167.3.338

[ref33] Lee H. S. (1990). Effect of pre-sowing seed treatments with GA_3_ and IAA on flowering and yield components in groundnuts. Korean J. Crop Sci. 35, 1–9.

[ref34] Li Z. Lv P. H. Du S. T. Ding J. Lv C. L. (2016). Effects of different types of plant growth regulators on the hypha growth of *Grifola frondose*. J. Northwest For. Univ. 31, 188–194.

[ref35] Li C. J. Nan Z. B. Li F. (2008). Biological and physiological characteristics of *Neotyphodium gansuense* symbiotic with *Achnatherum inebrians*. Microbiol. Res. 163, 431–440. doi: 10.1016/j.micres.2006.07.007, PMID: 16962754

[ref36] Liao Z. Suo Y. Xue C. Fu H. Wang J. (2018). Improving the fermentation performance of *Clostridium acetobutylicum* ATCC 824 by strengthening the VB_1_ biosynthesis pathway. Appl. Microbiol. Biotechnol. 102, 8107–8119. doi: 10.1007/s00253-018-9208-x, PMID: 29987383

[ref37] Luo Y. Guan Y. Q. Qi Z. X. Hao J. Z. Nuerziya Y. L. M. M. T. Wei P.. (2021). Effects of exogenous nutrition factors on mycelia growth of *Agaricus balchaschensis*. Xinjiang Agric. Sci. 58, 133–142.

[ref38] Ma M. Z. (2009). Biology, physiology and anti-fungal activities characteristics of *Neotyphodium lolii* of ryegrass. dissertation/master’s thesis. Lanzhou (Gansu): LanZhou University.

[ref39] Malinowski D. P. Belesky D. P. (2006). Ecological importance of *Neotyphodium* spp. grass endophytes in agroecosystems. Grassl. Sci. 52, 1–14. doi: 10.1111/j.1744-697X.2006.00041.x

[ref003] Mm A. Vm A. Fp B. Gi A. (2012). Fungal biodeterioration of historical library materials stored in Compactus movable shelves. Int. Biodeter. Biodegr. 75, 83–88. doi: 10.1016/j.ibiod.2012.03.011

[ref41] Naeem M. Bhatti I. Ahmad R. H. Ashraf M. Y. (2004). Effect of some growth hormones (GA_3_, IAA and Kintin) on the morphology and early or delayed iniation of bud of lintil (Lens culinaris Medik). Pak. J. Bot. 36, 801–809.

[ref42] Nambara E. Marionpoll A. (2005). Abscisic acid biosynthesis and catabolism. Annu. Rev. Plant Biol. 56, 165–185. doi: 10.1146/annurev.arplant.56.032604.144046, PMID: 15862093

[ref43] Nan Z. B. (1996a). Incidence and distribution of endophytic fungi in seeds of some native and introduced grasses in China. Acta Pratacul. Sin. 5, 1–8.

[ref44] Pope D. D. Hill N. S. (1991). Effects of various culture media, antibiotics, and carbon sources on growth arameters of *Acremonium coenophialum*, the fungal endophyte of *tall fescue*. Mycologia 183, 110–115. doi: 10.2307/3759839

[ref45] Ren G. M. Zhou M. L. Wu N. Gao Y. (2007). Influence of Vitamin B_1_ on several kinds of basidiomycete mycelia growth. J. Anhui Agric. Sci. 35, 8075–8076.

[ref48] Saikkonen K. Wäli P. Helander M. Faeth S. H. (2004). Evolution of endophyte–plant symbioses. Trends Plant Sci. 9, 275–280. doi: 10.1016/j.tplants.2004.04.005, PMID: 15165558

[ref50] Schardl C. L. Young C. A. Faulkner J. R. Florea S. Pan J. (2012). Chemotypic diversity of epichloae, fungal symbionts of grasses. Ecology 5, 331–344. doi: 10.1016/j.funeco.2011.04.005

[ref51] Schardl C. L. Young C. A. Hesse U. Amyotte S. G. Andreeva K. Calie P. J.. (2013). Plant-symbiotic fungi as chemical engineers: multi-genome analysis of the clavicipitaceae reveals dynamics of alkaloid loci. PLoS Genet. 9:e1003323. doi: 10.1371/journal.pgen.1003323, PMID: 23468653 PMC3585121

[ref52] Schulz B. Boyle C. Draeger S. Rommert A. K. Krohn K. (2002). Endophytic fungi: a source of novel biologically active secondary metabolites. Mycol. Res. 106, 996–1004. doi: 10.1017/S0953756202006342

[ref53] Siegel M. R. Latch G. C. M. Johnson M. C. (1987). Fungal endophytes of grasses. Annu. Rev. Phytopathol. 25, 293–315. doi: 10.1146/annurev.py.25.090187.001453, PMID: 22465162

[ref54] Škerget M. Kotnik P. Hadolin M. Hras H. R. Simonic M. Knez Z. (2005). Phenols, proanthocyanidins, flavones and flavonols in some plant materials and their antioxidant activities. Food Chem. 89, 191–198. doi: 10.1016/j.foodchem.2004.02.025

[ref55] Song Q. Y. Li F. Nan Z. B. Coulter J. A. Wei W. J. (2015a). Do *Epichloë* endophytes and their grass symbiosis only produce toxic alkaloids to insects and livestock? J. Agric. Food Chem. 68, 1169–1185. doi: 10.1021/acs.jafc.9b06614, PMID: 31922733

[ref001] Song M. L. Chai Q. Li X. Z. Yao X. Li C. J. Christensen M. J.. (2015b). An asexual Epichloë endophyte modifies the nutrient stoichiometry of wild barley (Hordeum brevisubulatum) under salt stress. Plant Soil. 387, 153–165. doi: 10.1007/s11104-014-2289-0, PMID: 23468653

[ref56] Spichal L. (2012). Cytokinins—recent news and views of evolutionally old molecules. Funct. Plant Biol. 39, 267–284. doi: 10.1071/FP11276, PMID: 32480780

[ref57] Tian P. Kuang Y. Nan Z. B. (2015). The characteristics of *Festuca sinensis* and its breeding potential. Pratacul. Sci. 32, 1079–1087.

[ref58] Tian P. Xu W. B. Li C. J. Song H. Nan Z. B. (2020). Phylogenetic relationship and taxonomy of a hybrid *Epichloë* species symbiotic with *Festuca sinensis*. Mycol. Prog. 19, 1069–1081. doi: 10.1007/s11557-020-01618-z

[ref59] Vázquez-de-Aldana B. R. García-Ciudad A. García-Criado B. Vicente-Tavera S. Zabalgogeazcoa I. (2013). Fungal endophyte (*Epichloë festucae*) alters the nutrient content of *Festuca rubra* regardless of water availability. PLoS One 8, 201–213. doi: 10.1371/journal.pone.0084539, PMID: 24367672 PMC3867530

[ref60] Wang M. N. (2019). Culture characteristics and resistance to metal ions *Epichloё* endophyte of *Festuca sinensis*. dissertation/master’s thesis. Lanzhou (Gansu): LanZhou University.

[ref62] Wang X. Y. Liu Y. Gao T. P. Xue L. G. Liu Y. B. Wan Z. D.. (2020). Effect of heavy-metal stress on fungal growth and pH of fermentation broth. Microbiology 47, 3226–3236. doi: 10.13344/j.microbiol.china.200678

[ref63] Wang X. H. Yi M. Liu H. Han Y. S. Yi H. L. (2016). Reactive oxygen species and Ca^2+^ are involved in cadmium-induced cell killing in yeast cells. Can. J. Microbiol. 63, 153–159. doi: 10.1139/cjm-2016-0258, PMID: 27995805

[ref64] Wei Y. K. Gao Y. B. Xu H. Su D. Zhang X. Wang Y. H. (2010). Occurrence of endophytes in grasses native to northern China. Grass Forage Sci. 61, 422–429. doi: 10.1111/j.1365-2494.2006.00551.x

[ref65] Weiler E. W. (1984). Immunoassay of plant growth regulators. Annu. Rev. Plant Physiol. 35, 85–89. doi: 10.1146/annurev.pp.35.060184.000505

[ref66] Xia C. Christensen M. J. Zhang X. X. Nan Z. B. (2018). Effect of *Epichloë gansuensis* endophyte and transgenerational effects on the water use efficiency, nutrient and biomass accumulation of *Achnatherum inebrians* under soil water deficit. Plant Soil 424, 555–571. doi: 10.1007/s11104-018-3561-5

[ref67] Yang Y. Chen N. Li C. J. (2011). The morphological diversity of endophytic fungal in *Festuca sinensis* in Gansu Province. Pratacult. Sci. 28, 273–278.

[ref68] Yang F. C. Ke Y. F. Kuo S. S. (2000). Effect of fatty acids on the mycelial growth and polysaccharide formation by *Ganoder malucidum* in shake flask cultures. Enzym. Microb. Technol. 27, 295–301. doi: 10.1016/S0141-0229(00)00213-1, PMID: 10899556

[ref69] You B. J. Lee H. Z. Chung K. R. Lee M. H. Huang M. J. Tien N.. (2012). Enhanced production of ganoderic acids and cytotoxicity of *Ganoderma lucidum* using solid-medium culture. Biosci. Biotechnol. Biochem. 76, 1529–1534. doi: 10.1271/bbb.120270, PMID: 22878212

[ref70] Young C. A. Hume D. E. Mcculley R. L. (2013). Forages and pastures symposium: fungal endophytes of tall fescue and perennial ryegrass: pasture friend or foe? J. Anim. Sci. 91, 2379–2394. doi: 10.2527/jas.2012-5951, PMID: 23307839

[ref71] Yuan Z. L. Dai C. C. Shi Y. Wang A. Q. Zhang D. Z. (2004). Study on the mechanism of endophytic fungus B3 promoting rice growth. Jiangsu Agric. Sci. 2, 10–13.

[ref72] Yuan Z. L. Zhang C. L. Lin F. C. (2010). Role of diverse non-systemic fungal endophytes in plant performance and response to stress: progress and approaches. J. Plant Growth Regul. 29, 116–126. doi: 10.1007/s00344-009-9112-9

[ref73] Yue Q. Miller C. J. White J. F. Richardson M. D. (2000). Isolation and characterization of fungal inhibitors from *Epichloë festucae*. J. Agric. Food Chem. 48, 4687–4692. doi: 10.1021/jf990685q, PMID: 11052720

[ref74] Zeng W. Qin W. Tian W. Xue Y. Liu S. (2015). Antioxidant activity in vitro of endophytic fungi from *Myricaria laxiflora*, a riparian plant with strong tolerance ability of flooding. J. Pure Appl. Microbiol. 9, 87–95.

[ref76] Zhang P. P. (2013). Characteristic of endophytic fungi isolated from Elymus and their effect on host resistances. Xinjiang Agricultural University, China. dissertation/master’s thesis. Urumchi (Xingjiang): Xinjiang Agricultural University.

[ref77] Zhang J. H. Wang C. L. Guo S. X. Chen J. M. Xiao P. G. (1999). Phytohormones produced by 5 endophytic fungi of orchidaceous medicinal plants. Acta Academiae Medicinae Sinicae, 49–54. 12567494

[ref78] Zhou L. Y. Li C. J. Zhang X. X. Johnson R. Bao G. S. Yao X.. (2015a). Effects of cold shocked *Epichloë* infected *Festuca sinensis* on ergot alkaloid accumulation. Fungal Ecol. 14, 99–104. doi: 10.1016/j.funeco.2014.12.006

[ref79] Zhu J. K. (2003). Regulation of ion homeostasis under salt stress. Curr. Opin. Plant Biol. 6, 441–445. doi: 10.1016/S1369-5266(03)00085-2, PMID: 12972044

